# 29. Not just skin deep: a case of neurological involvement in linear scleroderma en coup de sabre

**DOI:** 10.1093/rap/rky033.020

**Published:** 2018-09-20

**Authors:** Judith Jade, Marios Hadjivassiliou, Mohammed Akil

**Affiliations:** 1 Rheumatology, Sheffield Teaching Hospitals, Sheffield, UNITED KINGDOM; 2 Neurology, Sheffield Teaching Hospitals, Sheffield, UNITED KINGDOM

**Introduction:** Linear scleroderma en coup de sabre is a localised inflammatory disorder of the skin causing sclerosis in a linear distribution affecting the head and neck. We describe the case of a patient with progressive linear scleroderma en coup de sabre with unusual manifestations involving dental destruction and high T2 signal changes on magnetic resonance imaging (MRI) of the brain in the parenchyma below the area of scleroderma.

**Case description:** This 36 year old female first noticed an area of hair loss at 25 years old and was referred to dermatology and diagnosed with linear scleroderma en coup de sabre. She had no systemic features of connective tissue disease and antinuclear antibody testing, anticentromere antibody and extractable nuclear antigens were all negative. Despite topical treatment the area of linear scleroderma progressed and the patient was commenced on penacillamine. A year after her initial diagnosis, she developed an abnormality of her gums and teeth and maxillofacial review identified right upper incisor periodontal loss and radiograph confirmed local bone loss. Histopathology of the upper labial gingivae showed atrophied stratified squamous epithelia and chronically inflamed collagenous connective tissue in keeping with linear scleroderma. Pencillamine was later discontinued due to pregnancy, and the skin changes continued to spread causing disfigurement. The patient underwent psoralen and ultraviolet A radiation (PUVA) treatment and multiple reconstructive surgical procedures. Systemic disease modifying treatment was retried with oral methotrexate under the care of rheumatology but unfortunately with no efficacy and was therefore later withdrawn. The patient began suffering with headaches at the time of her initial presentation in 1997 and underwent an MRI brain scan which was unremarkable. She went on to develop diplopia which was thought to be due to enopthalmos secondary to the local involutional soft tissue changes around the orbit. The patient then underwent a further MRI scan in 2013 due to ongoing headaches and poor memory which revealed ipsilateral high T2 signal changes in the right corona radiata down to the lentiform nucleus and in the right internal capsule, below the area of linear sclerosis. Neurological investigations in the joint neurology and rheumatology clinic including computer tomography (CT) angiography have been unremarkable and it is felt this area of abnormality in the parenchyma is another manifestation of her linear scleroderma en coup de sabre. She has ongoing intermittent headaches but no further neurological symptoms. She has had a number of repeat MRI brain scans which show no progression of the parenchymal changes, and she remains under close monitoring with a multitude of specialities including rheumatology, neurology, dermatology, ophthalmology and plastic surgery.

**Discussion:** Linear scleroderma en coup de sabre is often considered to be a condition affecting the skin, in contrast to systemic sclerosis which has much broader manifestations in terms of other organ involvement. However this patient’s case highlights the fact that this condition is not just skin deep but can affect underlying soft tissue, the orbit, teeth, bone and brain parenchyma. Involution of the cranial bones and hemifacial atrophy is a documented complication of linear scleroderma but dental complications are rarely reported in the literature. Ipsilateral changes on brain imaging have previously been reported in patients with linear scleroderma. Patients may present with seizures, headache or other neurological manifestations such as hemiparesis. Case reports describe oligoclonal bands in the cerebrospinal fluid of patients with such radiological changes, pointing towards an underlying inflammatory pathology. Inflammation has been confirmed in one case report in which a patient underwent brain biopsy showing low grade localised vasculitis, however other case reports describe sclerosis with no inflammation on brain biopsy. Intravenous steroids and cyclophosphamide have been used to successfully treat progressive neurological complications, in terms of symptomatic and neuro-radiological improvement, again suggesting an inflammatory pathology.

**Key Learning Points:** This case highlights the importance of considering the wider manifestations of linear scleroderma en coup de sabre, including intracerebral pathologies when patients with this condition present with neurological symptoms and signs.

**Disclosure: J. Jade:** None. **M. Hadjivassiliou:** None. **M. Akil:** None.

